# Wearable coupled-quarter-mode SIW antenna platform with hybrid kinetic and ambient-light energy harvesting

**DOI:** 10.1038/s41598-023-32079-5

**Published:** 2023-03-27

**Authors:** Jelle Jocqué, Jo Verhaevert, Patrick Van Torre, Hendrik Rogier

**Affiliations:** Department of Information Technology (INTEC), IDLab, Ghent University-imec, Ghent, 9052 Belgium

**Keywords:** Devices for energy harvesting, Electrical and electronic engineering

## Abstract

As key enablers for smart fabric interactive textile (SFIT) systems, textile antenna systems and platforms need to be energy-efficient, low-profile and should guarantee a stable wireless body-centric communication link. Using multiple energy harvesters on and in the antenna platform is highly recommended to enable autonomous SFIT systems. Different sensors could be added to the system for monitoring the environmental and/or biophysical parameters of rescue workers, military personnel, and other safety workers. Therefore, a wearable coupled-quarter-mode (coupled-QM) substrate-integrated waveguide (SIW) antenna with optimally, seamlessly integrated hybrid kinetic and ambient-light energy harvesters is proposed. Two QM cavities are coupled via a non-resonant slot to create a compact antenna covering the [2.4; 2.4835] GHz Industrial, Scientific and Medical (ISM) band. The antenna platform fully consists of textile materials, being protective rubber foam and copper taffeta, enabling its unobtrusive integration into protective clothing. A novel, compact way of deploying a kinetic energy harvester inside the substrate, combined with flexible power management electronics on the antenna feed plane and a flexible ambient-light photovoltaic cell on the antenna plane, is proposed. The integrated antenna platform exhibits a measured impedance bandwidth of 307 MHz, a radiation efficiency of 88.57% and maximum gain of 3.74 dBi at 2.45 GHz. Wearing the antenna platform around a person’s wrist resulted in an average harvested power of 229.8 µW when walking in an illuminated room.

## Introduction

In the past few years, the growth of Internet of Things (IoT) devices and applications has seen an unknown surge. Most of these currently deployed devices are battery-powered and have an expected lifespan between 3 and 10 years. Shorter lifespans result in increased maintenance and higher operational costs, since engineers have to replace the batteries every few years or even substitute complete IoT devices. Battery-less IoT devices could considerably extend the lifespan of low-power IoT sensors, as most hardware components can last for decades while the lifetime of (rechargeable) batteries is typically only a few years. In comparison, supercapacitors have a lifespan ranging from 100,000 to 1,000,000 recharge cycles versus 500 to 10,000 recharge cycles for lithium-ion batteries. Replacing these bulky, polluting batteries with long-lasting supercapacitors can significantly increase the sustainability of IoT applications in the near future^1–3^.

Energy harvesting (EH) techniques will need to be exploited to recharge these supercapacitors. Energy can be collected from different sources in the environment, such as solar and radio-frequency (RF) energy. In body-centric applications, two additional energy sources that can be harvested, are thermal energy from body heat and kinetic energy from body movement.

In this paper, a novel technique is proposed to compactly integrate a kinetic energy harvester inside the substrate of a textile substrate-integrated waveguide (SIW) antenna. To the authors’ best knowledge, this is the first highly integrated wearable textile system that exploits both the inside and the outside of the antenna platform to seamlessly integrate active electronics and energy harvesters. In addition to the kinetic energy harvester inside the substrate, a flexible printed circuit board (PCB) with a power management system, a low-profile supercapacitor and a wireless ultra-low-power (ULP) microcontroller is designed for direct deployment on the antenna feed plane. In a final step, a flexible ambient-light photovoltaic panel is deployed directly on top of the antenna to enable hybrid EH. As a result, a wearable coupled-QM SIW antenna with optimally, seamlessly integrated hybrid kinetic and indoor light energy harvesters is proposed to enable autonomous and battery-less IoT devices and applications. For evaluation purposes only, a low-profile, flexible, lithium-ion polymer (LiPo) battery is added to the system. This battery is solely used in the validation process and is not a part of the power management system.

Similar antenna platform designs for integration into clothing were already developed in the past. A shoe-mounted antenna was proposed in combination with various EH techniques to localize moving subjects in a given area^4^. On the one hand, a wearable battery-free, active, paper printed radio-frequency identification (RFID) tag was designed on an organic flexible substrate including kinetic EH with a piezoelectric pushbutton. Its omnidirectional radiation pattern is not ideal for body-centric applications, albeit delivering a realized gain of 2 dBi and a fractional bandwidth of 10.8% in stand-alone conditions. On the other hand, a partially self-powered, health-monitoring and indoor localization shoe-mounted sensor module is presented. This module exploits a 5 cm by 8 cm rigid board with a piezoelectric transducer, a microcontroller unit and multiple transceivers. The antenna featured a more directional radiation pattern with a simulated realized gain above 7 dBi at 900 MHz when mounted on a shoe.

Two later publications realize a textile antenna for straightforward integration into the garments of the wearer. First, a textile SIW antenna topology, exhibiting high and stable radiation efficiency, is adopted as a platform for both energy harvesting and power management circuits^5^. The surface of the platform is exploited to integrate two off-the-shelf flexible solar cells, a thermoelectric generator (TEG) cell and a storage module. A disadvantage of this system is the connection to the TEG on the body via long wires. The platform could harvest up to 148.3 µW when worn by a dynamic wearer in an illuminated room at 17.5 $^\circ$C. In this paper, a smaller and more integrated antenna platform is obtained by exploiting a bandwidth enhancement technique^6^, on the one hand, and directly integrating a kinetic energy harvester inside the antenna substrate, on the other hand. Next, a wearable textile antenna RFID tag with sensing, processing, and decision-taking capability, is described in literature^7^. A shorted circular patch antenna with a monopolar radiation pattern is leveraged as antenna topology and the flexible solar cell is patterned to fit to the antenna patch contours. It was placed in such a way that the radiating edge remains uncovered. As a result, a more integrated wearable antenna platform for RFID purposes is realized, but with only a single form of EH, making the proposed system unsuitable for more demanding IoT devices and applications.

More recently, a new type of antenna, capable of harvesting radio frequency, vibration and light energy, based on piezoelectric and solar film materials, is found in literature^8^. The antenna relies on a stacked circular patch topology implemented on a Polyvinylidene fluoride (PVDF) substrate with a high dielectric constant of $\epsilon _r~=~9.5$. The platform was able to generate an output voltage of 1.5 V under a 75 Hz vibration excitation, but it is unsuitable for body-centric applications, because body movement exhibits a much lower frequency range.

To increase the kinetic EH, other technologies of mechanical harvesting should be exploited to enable autonomous wireless systems solely powered with EH techniques. There are two popular categories of kinetic energy harvesting devices, being piezoelectric generators and electromagnetic generators. The size of the electromagnetic generator is inversely proportional to its resonant frequency^9^. Since body movement typically exhibits a resonance frequency range from 0 to 20 Hz, it is difficult to develop and deploy compact EH devices on the body due to the correspondingly larger size of the electromagnetic generator^10^. It is also stated that electromagnetic generators generally produce a high output current at a lower nominal voltage, which makes them more suited for EH applications.

Kinetron (kinetron.eu), a manufacturer specialized in motion-based energy harvesting systems, develops micro generator systems (MGS), customized to power autonomous applications^11^. The MGS 32.8, one of their most recently developed micro generators, is exploited in this paper. In the past, an older series of micro generator systems (MGS 26.4) has been mentioned in several publications^12–16^. Recently, a kinetic harvesting circuit was designed that could harvest up to 1.1 J per day with a passive voltage doubler^12^. This was improved to 1.4 J per day with a negative voltage converter (NVC)^13,14^. Both papers assumed two hours of walking and 30 minutes of running per day. A practical application, a perpetual wearable camera acquisition system, was also designed with a passive voltage doubler and powered by an MGS 26.4 harvester^15^. Experiments showed a frame rate of 15 frames per hour when saving images to an external SD card. Previously mentioned papers were primarily focused on the design of the energy harvesting circuit and conversion circuit. Moreover, Mayer et al.^16^ concentrate more on the design and architecture of a smart power unit to lower the energy consumption of the application in different power modes and scenarios. In addition, a type of piezoelectric generator is used to extend the lifetime of a self-powered smart basketball module^17^. Unfortunately, no specific data were provided to indicate the amount of increased battery life and to compare this to electromagnetic generators.

To integrate the antenna platform with built-in EHs in the garments of the user, an SIW cavity-backed slot antenna topology is adopted in this paper. An improved fabrication methodology, also applied in this paper, was recently proposed to implement foldable all-textile cavity-backed slot antennas^18^. In order to create a compact antenna platform for integration into the garments of the user, miniaturization techniques have to be exploited. By bisecting the SIW cavity once or multiple times along its virtual quasi-magnetic walls, half-mode (HM) or even quarter-mode (QM) cavities can be created^19–22^. A wideband HMSIW cavity-backed slot antenna on cork substrate and a coupled-HM cavity-backed slot antenna in air-filled SIW technology were already presented^23,24^. Later, a miniaturized wearable antenna in textile materials that relies on QM SIW topology was proposed^25^. Since then, more rigid antenna structures based on QM SIW topology were introduced for wideband and dual-band antennas in next-generation wireless systems^26–30^.

Section “Antenna as integration platform” outlines the design aspects, materials, topology and operation principle of the textile coupled-QM SIW antenna platform. The system architecture, including the circuit design and the realization of the flexible PCB, is described in detail in section “System architecture”. Next, simulation and measurement results of both the antenna platform and energy harvesting are discussed in section “Simulation and measurement results”, which ends with an example application scenario. Finally, a conclusion is formulated in the final section of this manuscript.

## Antenna as integration platform

In this section, more specific details are provided regarding the antenna design. Specifically, the antenna will be used as an integration platform for energy harvesting and, therefore, several design requirements are imposed, followed by the antenna materials, the antenna topology and the operation principle.

### Design aspects

A low-profile, compact and flexible textile antenna is designed for body-centric wireless communication in compliance with the IEEE 802.15.4 standard. The antenna topology is selected such that the integration of a kinetic EH module inside the antenna and an ambient-light photovoltaic cell on the antenna plane has limited influence on its radiation characteristics and performance. To operate in the [2.4; 2.4835] GHz Industrial, Scientific and Medical (ISM) frequency band, it is required that the magnitude of the antenna reflection coefficient with respect to 50 $\Omega$ remains below -10 dB in the entire frequency band. Furthermore, since the antenna consists of different flexible materials, it must be able to operate under different degrees of deformation. Moreover, the electromagnetic properties, such as the relative permittivity $\epsilon _r$ and the loss tangent tan $\delta$, may differ between different sheets of synthetic rubber, applied as antenna substrate. Consequently, small variations in $\epsilon _r$ should have no impact on the antenna performance. Since the antenna is intended for application in off-body wireless communication, in addition, a minimal front-to-back-ratio (FTBR) of 6 dB is imposed to minimize the absorption of radiation by the human body. Finally, a high radiation efficiency is beneficial in terms of system autonomy when exploiting the antenna platform in a low-power application solely powered by harvested kinetic and/or ambient-light energy.

### Antenna materials

To comfortably integrate this antenna platform into the garments of the user, dedicated textile materials are selected to manufacture a mechanically flexible antenna. The dielectric substrate was carefully chosen from a wide range of closed-cell expanded rubbers by Interep (interep.fr), suitable for integration as protective foams in garments. More than 30 different types of these closed-cell expanded rubbers were characterized by a resonating method^31^ for the [2.4; 2.4835] frequency band. Next, the different types of rubbers were compared on the basis of their dielectric permittivity, loss tangent, dimension stability and compression set resistance^32^. The type of rubber selected for this application is an ethylene propylene diene monomer (EPDM) with $\epsilon _r$ = 1.3 and tan $\delta$ = 0.001, which features low water absorption, high temperature resistance and excellent compression set resistance without compromising flexibility^33^. Such types of closed-cell rubbers are typically used as protective foam in firefighter jackets. The conductive material used for this antenna is a Pure Copper Polyester Taffeta Fabric by Less EMF (lessemf.com), with a thickness of 0.08 mm and a very low sheet resistivity of 0.05 $\Omega$/sq^34^.

### Antenna topology and operation principle

To comply with the design specifications, an SIW cavity-backed slot antenna topology is selected because of its excellent radiation efficiency, low fabrication cost, low-profile and ease to integrate active electronics on the feed plane of the antenna. A disadvantage of the cavity-backed slot antenna topology is its narrowband nature due to excitation of a single resonant cavity mode^35^. To meet the requirements with wide margins at both ends of the -10 dB impedance bandwidth in a small form factor, a bandwidth enhancement technique is exploited^6^.

**Figure 1:**
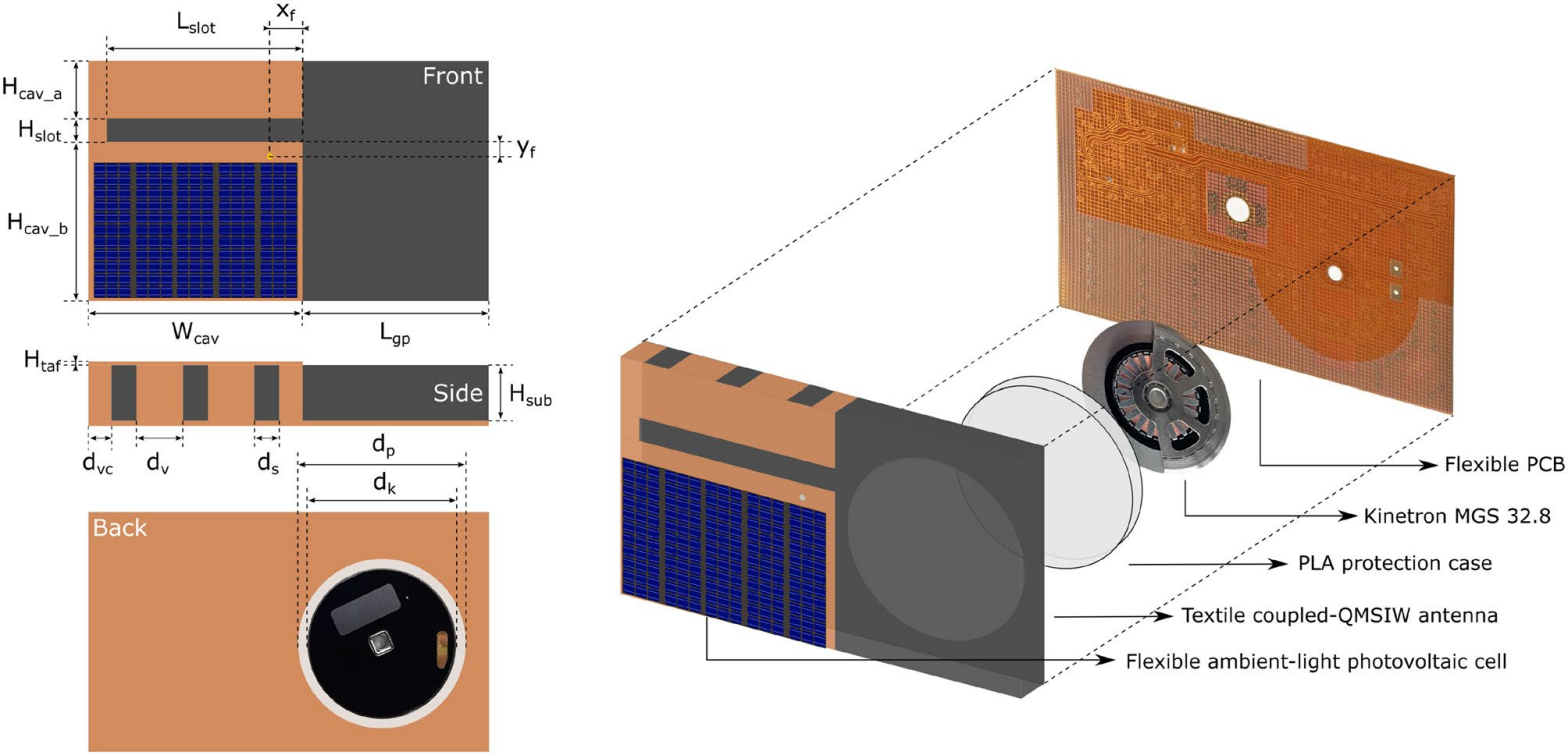
Proposed coupled-QM SIW antenna topology with flexible ambient-light photovoltaic cell integrated on top and Kinetron MGS 32.8 integrated inside the substrate, top view, side view and bottom view on the left hand side, and in 3D on the right hand side (H_cav_a_ = 12 mm, H_cav_b_ = 34 mm, H_slot_ = 5 mm, H_taf_ = 0.08 mm, H_sub_ = 12 mm, W_cav_ = 46 mm, L_slot_ = 30 mm, L_gp_ = 40 mm, X_f_ = 7 mm, Y_f_ = 3 mm, d_v_ = 10 mm, d_vc_ = 5 mm, d_s_ = 5.33 mm, d_k_ = 32.4 mm, d_p_ = 36 mm).

The proposed topology for the coupled-QM SIW antenna is depicted in Fig. 1. First, on the top plane of the antenna, a flexible ambient-light photovoltaic cell is deployed directly on the copper Taffeta with limited impact on the antenna performance. Second, this proposed topology enables the integration of a kinetic EH module inside the antenna substrate. By increasing the antenna ground plane along the right-hand side of the top view, the FTBR is increased, on the one hand, and the module is integrated sufficiently far away from both QM antenna cavities, on the other hand. As a result, the kinetic EH module is inserted in an area of the substrate where almost no electromagnetic fields are present^23^.

**Figure 2:**
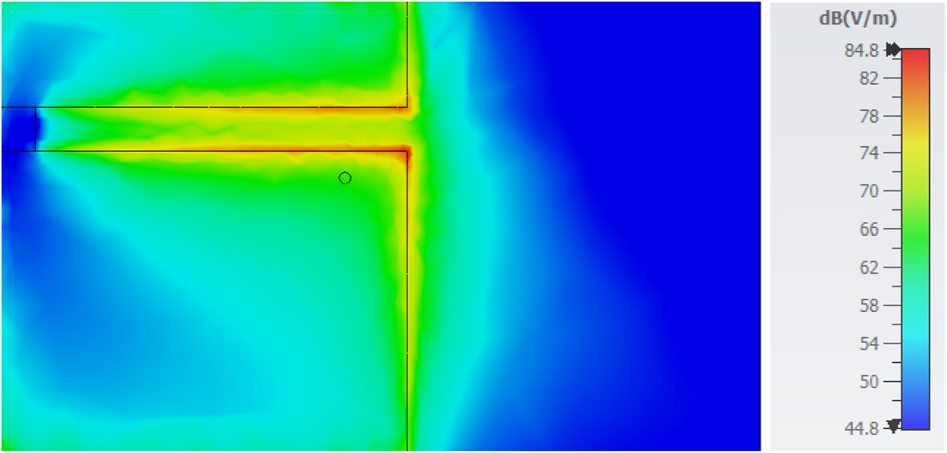
Simulated electric field distribution observed inside the resonant cavities at 2.45 GHz in dBV/m.

In the proposed topology, depicted in Fig. 1, two QM SIW resonant cavities are coupled via a non-resonant slot to create a broader bandwidth. The top cavity planes and the feed plane are connected by conducting Taffeta vertical cavity walls folded around the textile substrate^18^. A large fractional impedance bandwidth is achieved by dimensioning both cavities such that both excited hybrid modes are close to each other in the frequency band of interest. Each miniaturized QM SIW cavity in Fig. 1 exhibits a fundamental resonant frequency, calculated by (2) in^26^. When the QM SIW resonators are brought in close proximity, mode bifurcation due to the coupled resonating cavities occurs. By carefully tuning the dimensions of both QM SIWs and optimizing the coupling between them, their resonance frequencies are brought in each other’s proximity. As such, the two resonance peaks in the frequency spectrum are combined in one broad resonance peak around 2.45 GHz. The normalized electric field distribution at the center frequency is depicted in Fig. 2. The coaxial probe, feeding the lower cavity, is positioned such that also the upper cavity mode is excited through parasitic coupling. Its position also influences the impedance matching and has to be carefully determined.

**Figure 3:**
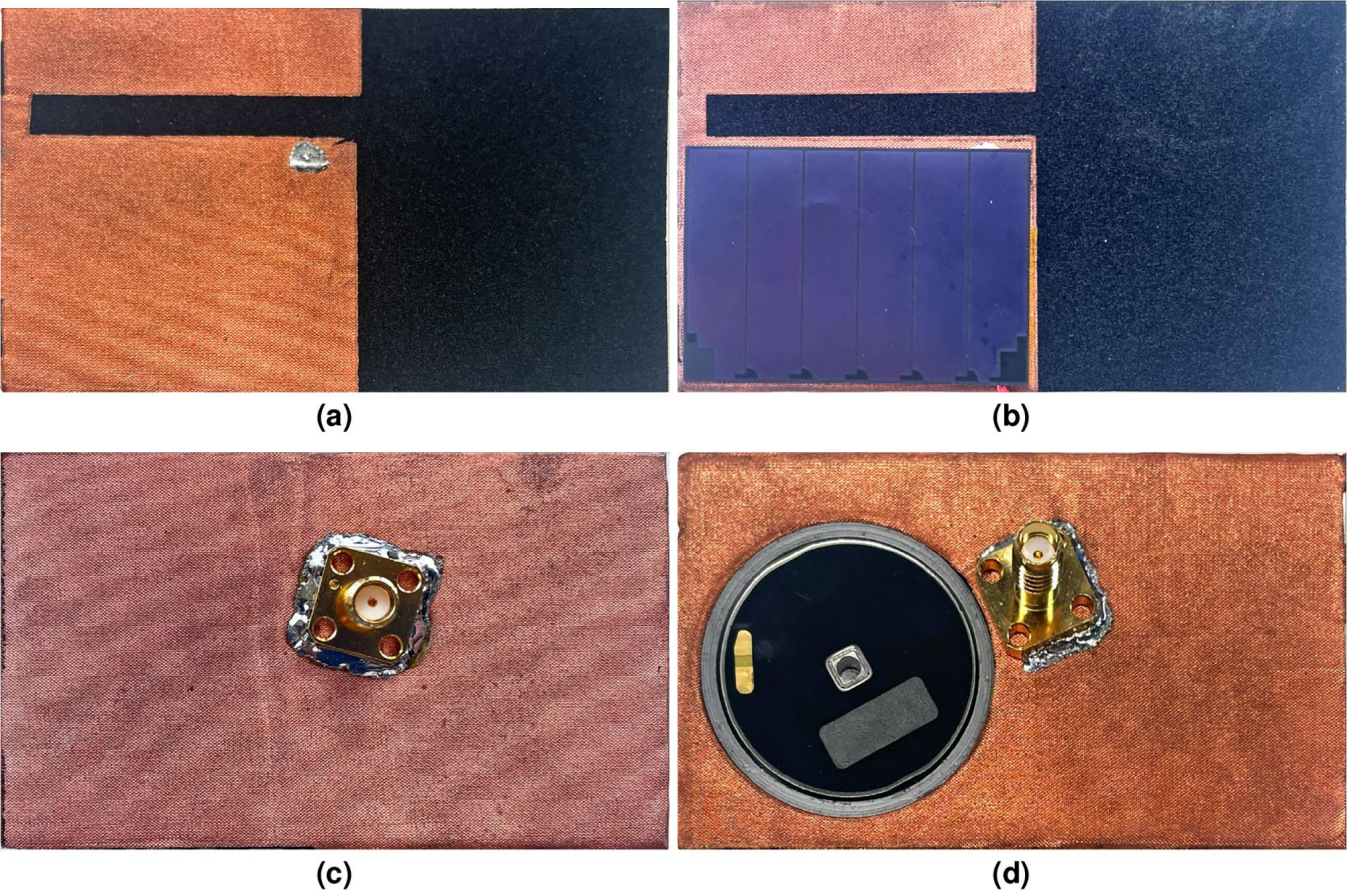
Fabricated antenna top view without photovoltaic cell **(a)** and with photovoltaic cell **(b)**, and bottom view without kinetic EH module **(c)** and with kinetic EH module **(d)**.

First, all antenna dimensions were optimized for operation in free space stand-alone conditions without the integration of energy harvesters and active electronics, using CST Microwave Studio. Second, the antenna performance was further fine-tuned for resilient operation with integrated energy harvesters and active electronic circuits deployed in its close proximity. Finally, the stability of the antenna characteristics was validated by adding a biological model of a representative human body to the simulation environment, verifying the requirements discussed in subsection “Design aspects”. This body model was imported via the *Voxel Data Import*, provided by CST Microwave Studio, and is a very accurate representation of an 38 years old male person with a height of 176 cm and a weight of 69 kg. The fabricated antennas with and without energy harvesters are shown in Fig. 3.

## System architecture

### Circuit design

**Figure 4:**
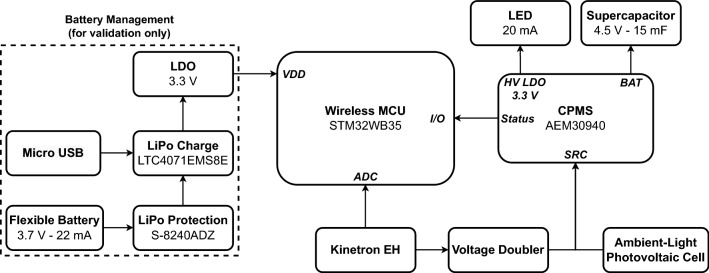
High-level block diagram of the proposed hybrid energy harvesting and power management circuit.

A high-level block diagram of the circuit is depicted in Fig. 4. The circuit consists of three major building blocks, being the wireless microcontroller unit (MCU), the central power management system (CPMS) and the battery management circuit.

First, an MGS 32.8 kinetic energy harvester by Kinetron, with a diameter of 32.8 mm and a thickness 4 mm, is used to generate power from body movement. This device is seamlessly integrated inside the antenna substrate. The generator is connected to the CPMS via a voltage doubler composed of BAT54HT1G Schottky diodes by ON Semiconductor^36^. The diodes are selected based on their extremely low forward voltage drop of 220 mV for a forward current of 0.1 mA, high reverse voltage of 30 V and compact footprint with a SOD-323 package. Second, a thin, lightweight, and flexible ambient-light photovoltaic cell is deployed directly on top of the antenna plane^37^. This photovoltaic cell is also connected to the input of the CPMS.

A recent and highly efficient ambient energy manager IC, AEM30940, is applied as the core of the CPMS^38^. This IC was found to be the most suited after an extensive comparison between different power management ICs that are currently on the market. It outperforms other ICs in terms of cold start input voltage, operating quiescent current and continuous EH voltage. It also features two highly efficient low-dropout (LDO) regulators to directly power a low-power microcontroller, which makes the IC perfectly suited for battery-less autonomous applications. The CPMS relies on a low-profile supercapacitor as a primary energy storage with a capacitance of 15 mF, a rated voltage of 4.5 V, and a maximum leakage current of 5 µA^39^. Finally, a simple LED with a constant current consumption of 20 mA is used to discharge the supercapacitor and to emulate the energy consumption in different use cases.

During the verification process, a small and flexible lithium-ion polymer (LiPo) battery is added to provide additional power to the microcontroller for the validation measurements and the logging of experimental data only^40^. A special LiPo protection and charge circuit was designed to support this type of battery. The micro-USB port is used to recharge the battery and a 3.3 V LDO is added to power the MCU. The flexible battery is not a part of the CPMS and only serves as an external power source for the microcontroller to log measurement data. The supercapacitor is the primary energy source of the CPMS.

### Flexible PCB design

In section “Antenna as integration platform”, the design aspects, topology and materials of the developed antenna were discussed. To guarantee fully autonomous antenna operation in multiple use cases, the antenna must be designed such that, besides providing its primary communication functionality, it simultaneously serves as a flexible integration platform for kinetic and ambient-light EHs. Therefore, a flexible PCB was developed that is suitable for integration onto the feed plane of the antenna while offering connections to the MGS 32.8 inside the antenna and the ambient-light photovoltaic cell on top of the antenna plane. The flexible PCB measures 85 mm $\times$ 50 mm $\times$ 161 µm, customized to size of the antenna feed plane (86 mm $\times$ 51 mm) and is composed of a flexible polyimide (PI) foil of 25 µm, a top and bottom copper layer of 18 µm and a top and bottom PI cover layer (25 µm) with an adhesive layer (25 µm). Special care was taken when designing the board to provide as much flexibility as possible without compromising its functionality. General guidelines, such as curved traces with a large bend radius and teardrop vias, were applied to strengthen the traces. Hatched reference planes are used on both the top and bottom layers instead of rigid copper planes to provide extra flexibility. Hatching the reference planes has little impact on the functionality of the PCB.

An additional footprint was added on the top plane of the flexible PCB to place a SubMiniature Version A (SMA) coaxial connector that is connected to the antenna for measurement purposes. The bottom reference plane of the flexible PCB is partially covered by a solder mask to connect it to the feed plane of the antenna through conductive glue or double-sided copper tape.

**Figure 5:**
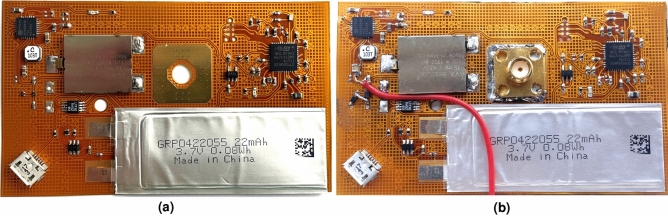
Realized flexible PCB stand-alone **(a)** and attached to antenna feed plane **(b)**.

Finally, the cathode of the photovoltaic cell is connected by a wire to the input of the CPMS IC. This wire is carefully routed along one of the folded vertical cavity walls without disturbing the antenna operation. The anode of the photovoltaic cell is taped to the top side of the antenna with double-sided copper tape and thereby also connected to the reference port of the CPMS. The flexible PCB itself is also taped to the bottom of the antenna and, as a result, the reference of the photovoltaic cell is connected to the reference of the flexible PCB. The realized stand-alone flexible PCB with all the components is depicted in Fig. 5a and attached to the antenna feed plane in Fig. 5b.

## Simulation and measurement results

In this section the antenna simulations and measurements, and the performance of the energy harvesting and power management module are discussed. All experimental protocols in this paper were approved by the ethical committee of the Faculty of Engineering and Architecture of Ghent University. Furthermore, the authors declare that all experiments were carried out while following the relevant guidelines and regulations. Moreover, informed consent has also been obtained from all participants, of the study.

### Antenna simulations and measurements

**Figure 6:**
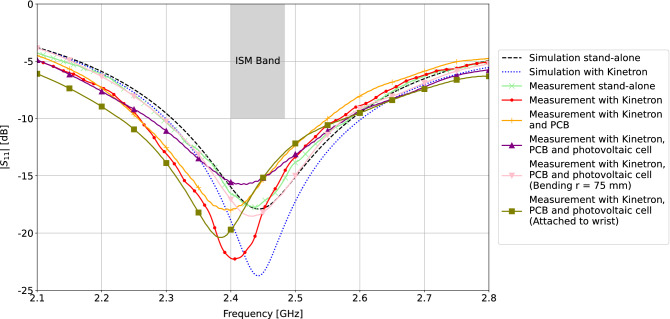
Simulated and measured reflection coefficient $|S_{11}|$ (dB) versus frequency, with and without Kinetron and photovoltaic cell. Simulation stand-alone free space, no harvesters nor electronic components present (full line), and with Kinetron (dashed line); measurement stand-alone free space, no harvesters nor electronic components present (marker ‘times’), and with Kinetron (marker ‘dot’), with Kinetron and PCB (marker ‘plus sign’), with Kinetron, PCB and photovoltaic cell (marker ‘upward triangle’), with Kinetron, PCB and photovoltaic cell under bending in azimuth with r = 75 mm (marker ‘downward triangle’), and with Kinetron, PCB and photovoltaic cell when attached to the wrist (marker ‘square’).

Reflection coefficient measurements were conducted with a Keysight N5247A PNA-X Microwave Network Analyzer. Figure 6 shows the simulated and measured input reflection coefficients for the developed antenna both in stand-alone conditions, being operation in free space without any electronics deployed near the antenna, and also with integrated energy harvesters and active electronics. Simulation results show that integrating a micro generator inside the antenna substrate has a positive effect on the impedance bandwidth, resulting in a bandwidth of 315 MHz (12.9%). The achieved bandwidth covers the entire [2.4; 2.4835] GHz frequency band and provides margins of 100 MHz at both sides of the spectrum, as was envisaged in the design requirements in subsection “Design aspects”. Verification of the complete integrated antenna platform was performed with extra measurements by including the flexible PCB and photovoltaic cell. All measurement results are in line with simulations but they are slightly shifted to the left due to manual fabrication of the antenna, leading to a lower resonance frequency. The measured realized impedance bandwidth for the fully integrated antenna platform amounts to 307 MHz, covering a frequency band from 2.274 GHz to 2.581 GHz, resulting in a fractional bandwidth of 12.5%. In addition, the resilience of the antenna performance to bending conditions is important for wearable applications. The fully integrated antenna platform was measured under cylindrical bending in the azimuth plane with a bending radius of 75 mm. The result of the bending test has been added to Fig. 6, showing limited impact on the impedance bandwidth. Finally, the reflection coefficient was measured of the fully integrated antenna platform when worn around a person’s wrist. This resulted in a bandwidth of 347 MHz (14.2%), covering the entire bandwidth in the envisioned application.

**Figure 7:**
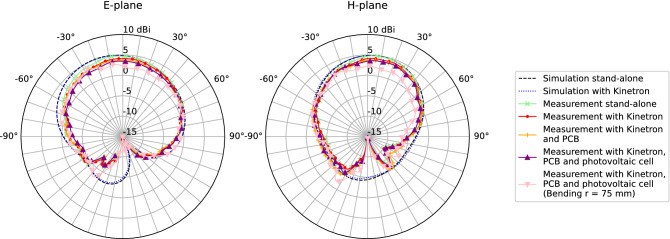
Measured versus simulated radiation pattern at 2.45 GHz, both in E-plane ($\phi$ = 0$^\circ$) and H-plane ($\phi$ = 90$^\circ$). Simulation stand-alone free space, no harvesters nor electronic components present (dashed line), and with Kinetron (dotted line); measurement stand-alone free space, no harvesters nor electronic components present (marker ‘times’), with Kinetron (marker ‘dot’), with Kinetron and PCB (marker ‘plus sign’), with Kinetron, PCB and photovoltaic cell (marker ‘upward triangle’), and with Kinetron, PCB and photovoltaic cell under bending in azimuth with r $=$ 75 mm (marker ‘downward triangle’).

**Table 1:** Simulated and measured frequency-domain characteristics.

Parameters	Simulation		Measurement				
	(a)	(b)	(a)	(b)	(c)	(d)	(e)
Gain (dBi)	5.22	5.14	4.95	4.21	3.93	3.74	3.57
Efficiency (%)	99.39	99.32	97.54	92.74	92.45	88.57	82.21
HPBW ($^\circ$)							
Azimuth	95	99	82.2	89.7	98.7	97	120.6
Elevation	125.6	124.9	116.3	127	130.6	126.8	149.9

(a) Stand-alone free space, no harvesters nor electronic components present, (b) kinetron, (c) kinetron and flex PCB, (d) kinetron, flex PCB and photovoltaic cell. (e) Kinetron, flex PCB and photovoltaic cell (bending r = 75 mm).

The far-field performance of the antenna platform was verified inside an anechoic chamber with outer dimensions of 8 m by 4 m by 4 m, using a Keysight N5247A PNA-X Microwave Network Analyzer with a NSI-MI spherical near-field scanner system. Figure 7 shows the simulated and measured far-field gain patterns in the E-plane and H-plane, respectively. Table 1 summarizes these far-field results for different integrated components. The coaxial cable attached to the connector on the feed plane of the antenna was covered by absorbers to minimize cable effects.

The simulated FTBR in stand-alone conditions (free space, no harvesters nor electronic components present) amounts to 9.27 dB and to 9.73 dB when the MGS 32.8 EH is inserted into the substrate. In stand-alone conditions (a), the measured maximum gain of 4.95 dBi and gain pattern shape correspond well to the simulation results. Integration of the MGS 32.8 EH (b) results in a slightly lower maximum gain and efficiency in measurements due to the approximate model used in simulations for the kinetic harvester. Next, measurements performed with the flexible PCB attached to the feed plane of the antenna platform (c) show an additional decrease in realized gain and efficiency due to some additional losses in the polyimide substrate. In the measurement with the ambient-light photovoltaic cell attached on top of the lower cavity on the antenna plane (d), again a small decrease in realized gain and efficiency has occurred. This can be explained by a slightly higher reflection coefficient in Fig. 6, because the photovoltaic cell is attached on top of the feeding pin and has a small impact on the impedance matching. In a final measurement, the antenna platform, with the kinetic EH, the flexible PCB and the ambient-light photovoltaic cell, was measured under cylindrical bending in the azimuth plane (e). All requirements of the complete integrated antenna platform are fully met under bending, with a realized gain of 3.74 dBi and a total efficiency of 88.57%. The half-power beam width (HPBW) amounts to 97$^\circ$ and 126.8$^\circ$ in the azimuth plane and elevation plane, respectively.

**Figure 8:**
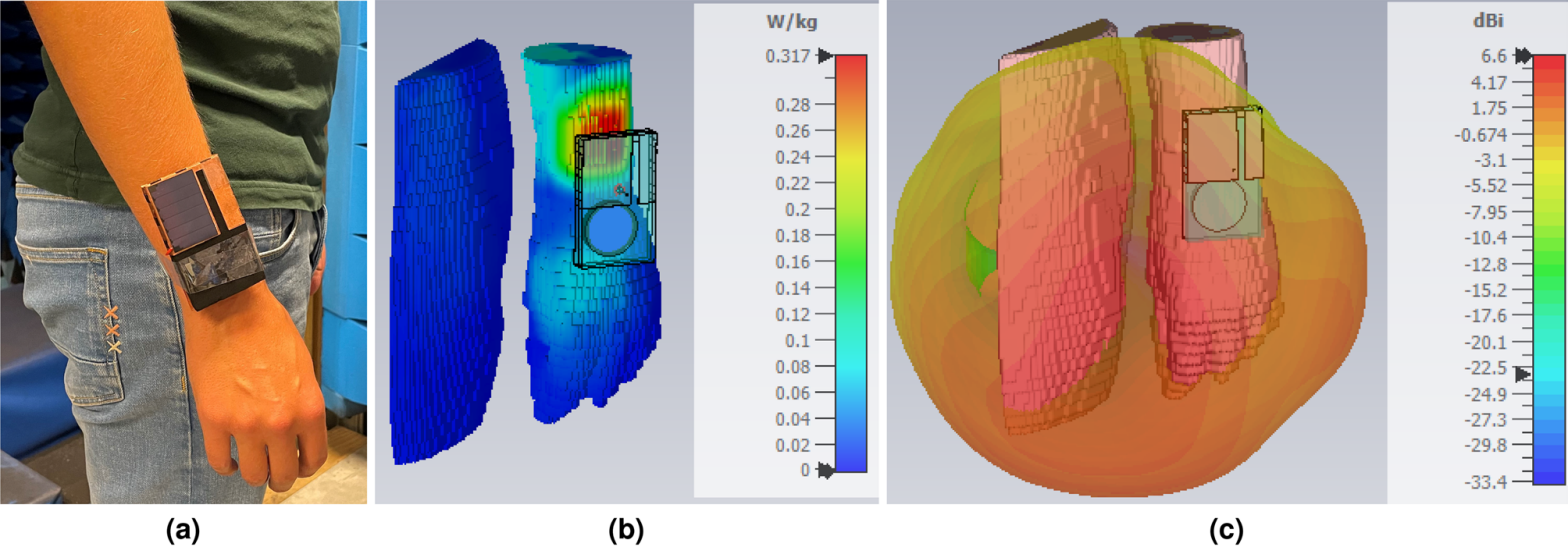
Antenna platform worn around a person’s wrist **(a)**, simulated SAR according to the IEEE C95.1 standard in front of a real biological model of a wrist at 2.45 GHz **(b)** and radiation pattern in front of a real biological model of a wrist at 2.45 GHz **(c)**.

Lastly, the on-body antenna performance was analyzed. The specific absorption rate (SAR) limits were verified, according to the IEEE C95.1 standard (United States) with a maximum value of 1.6 W/kg when averaged over 1 g of tissue, and according to the IEC 62209-1 standard (European Union) with a maximum value of 2 W/kg when averaged over 10 g of tissue. For this purpose, a real biological model of a wrist was used in combination with the proposed wearable antenna platform. SAR results, depicted in Fig. 8b, show a maximum value of 0.53 W/kg according to the IEEE C95.1 standard and a maximum value of 0.32 W/kg according to the IEC 62209-1 standard. Both simulated maximum SAR values remain well below the limit. In addition, the radiation pattern was simulated in the presence of the same biological model at 2.45 GHz, shown in Fig. 8c. The obtained radiation pattern slightly deviates from that of free space due to the presence of the human body, with a realized gain of 6.6 dBi and an efficiency of 86.84%. These simulated and measured radiation patterns demonstrate that the proposed antenna system is highly suited for off-body communication applications in compliance with the IEEE 802.15.4 standard.

Table 2 compares the proposed antenna platform to other reported antenna platforms in literature that are suitable for energy harvesting. Note that the height of the proposed system platform is larger than for the other reported antenna systems. This is because the current design has been optimized in terms of dimensions and antenna topology for integration of an off-body communication antenna with kinetic harvester into protective pads that are deployed on human arms (elbows/wrists) or legs (knees/ankles), which exhibit the most movement. Thereby, the relatively thick foam substrate of the pads simultaneously offers protection to the wearer and to the kinetic harvester. In contrast, Lemey et al.^5,7^ propose antenna topologies on thinner substrates, but with larger lateral dimensions, which are more suited for deployment on flatter parts of the body, such as torso, back and shoulders. The antenna proposed in^8^ has not been optimized for operation when deployed on a human body.

**Table 2:** Comparison of the proposed antenna platform with other reported antenna platforms suitable for energy harvesting.

Refs	Frequency (GHz)	Substrate	Size (l $\times$ w $\times$ h) (mm) ($\lambda$)	Bandwidth ([MHz])	Gain (dBi)	Efficiency (%)
Ref.^5^	2.45	Polyurethane	126.5 $\times$ 131.5 $\times$ 4 (1.03$\lambda$ $\times$ 1.07$\lambda$ $\times$ 0.03$\lambda$)	409	4.7	–
Ref.^7^	2.45	Polyurethane	113 $\times$ 66 $\times$ 4 (0.92$\lambda$ $\times$ 0.54$\lambda$ $\times$ 0.03$\lambda$)	150	2.7	65
Ref.^8^	2.45	Polyvinylidene fluoride	53 $\times$ 60 $\times$ 0.6 (0.43$\lambda$ $\times$ 0.5$\lambda$ $\times$ 0.005$\lambda$)	1000	3	–
This work	2.45	EPDM	51 $\times$ 86 $\times$ 12 (0.42$\lambda$ $\times$ 0.7$\lambda$ $\times$ 0.1$\lambda$)	307	3.74	88.57

### Performance of the energy harvesting and central power management system

#### Central power management system

The AEM30940 IC in the CPMS is used to extract DC power from the MGS 32.8 and the flexible ambient-light photovoltaic cell. The IC features a cold start input voltage and power of only 380 mV and 3 µW. The open-circuit voltage ($V_{oc}$) of the ULP boost regulator, used to charge a supercapacitor, senses for its Maximum Power Point Tracking (MPPT) every 0.33 s. The voltage level of the MPP, $V_{mpp}$, depends on the input power available at the source. The ratio $V_{mpp}$/$V_{oc}$ can be configured to 50%, 65% and 80%. Preliminary measurements, performed on the antenna platform with the MGS 32.8 and photovoltaic cell attached to the AEM30940, provide the best results for a $V_{mpp}$/$V_{oc}$ ratio of 80%. This is the configuration used in the real-life indoor measurement scenarios, discussed in subsection “Performance of the proposed energy harvesting and power management module in a real-life indoor environment”. Two internal LDOs can be used simultaneously when the supercapacitor is sufficiently charged. The low-current LDO can provide a load current up to 20 mA, while the high-current LDO can provide a load current up to 80 mA with a 300 mV drop-out.

#### Integrated micro generator system

**Figure 9:**
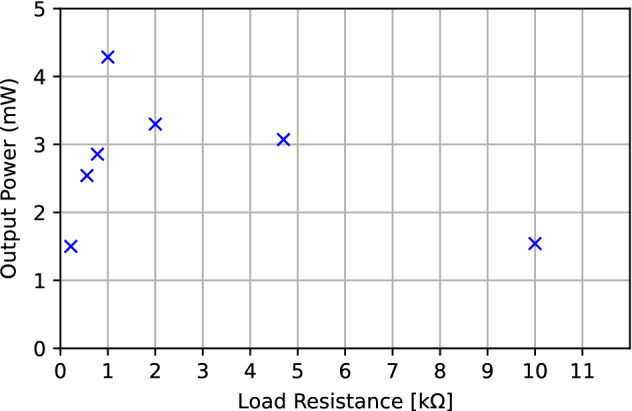
Output power generated by a half spin of the MGS 32.8, as a function of load resistance.

The output power generated by a half spin of the MGS 32.8 as a function of the load resistance is shown Fig. 9. Measurements were repeated 10 times for every load and averaged to obtain the results in Fig. 9^12^. In addition to the calculation of the average output power generated by a half spin of the MGS 32.8, the average energy of the waveform was also determined. For the optimal load value of 1 k$\Omega$, the output waveform yields an average output power of 4.286 mW, an average duration of 37 ms and as a result a total energy of 1.5 mJ.

#### Flexible ambient-light photovoltaic cell deployed on the antenna platform

**Figure 10:**
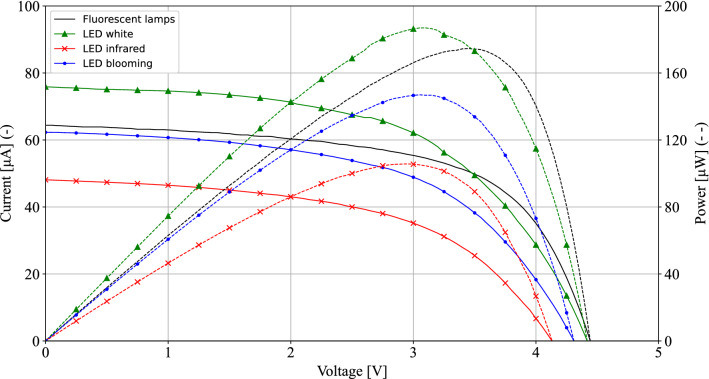
DC I–V (solid lines) and P–V (dashed lines) characteristics of the flexible ambient-light photovoltaic cell, when illuminated with fluorescent lamps and different types of LED lights. Fluorescent lamps (no marker), LED white (marker ‘upward triangle’), LED infrared (marker ‘times’), LED blooming (marker ‘dot’).

A 2450 source measure unit (SMU) by Keithley was used to obtain the DC I–V and P–V characteristics of the flexible ambient-light photovoltaic cell. The photovoltaic cell was illuminated by fluorescent lamps, used in the real-life indoor measurement scenarios, which are discussed in subsection “Performance of the proposed energy harvesting and power management module in a real-life indoor environment”, and three types of 20 W LED lights. The white LED has a color temperature of 6500 K, while the infrared LED has a color temperature of 2100 K combined with 730 nm red light and the blooming LED is a combination of 6500 K, 2100 K and 660 nm red light. Measurements are performed in a dark room with the different light sources mounted at a height of 150 cm above the photovoltaic cell. The DC I–V and P–V characteristics are depicted in Fig. 10. The white LED light source yields the highest measured maximum power point of 186.9 µW for a voltage of 3.01 V. The maximum power point measured with the fluorescent lamps, amounts to 174.6 µW for a voltage of 3.45 V. Since, the flexible photovoltaic cell provides a $V_{oc}$ of 4.2 V, the measurements provide an optimal $V_{mpp}$/$V_{oc}$ ratio of 82% for the real-life indoor measurements.

### Performance of the proposed energy harvesting and power management module in a real-life indoor environment

All measurements were performed in an indoor office environment to demonstrate the energy harvesting potential when the antenna platform is worn as a wearable sensor node around a person’s wrist, as demonstrated in Fig. 8a. The following three experiments were executed to verify the potential to scavenge energy in different situations:

1. Ambient-Light Scenario—a static wearer in an illuminated room;
2. Body Movement Scenario—a dynamic wearer in a nonilluminated room;
3. Hybrid Scenario—a dynamic wearer in an illuminated room.

Every measurement scenario extended over a time span of 1 min and was repeated ten times to provide more accurate results. The three scenarios were performed by a male test person of 1m80 in height in a 2.9-m high room, illuminated by fluorescent lamps mounted at the ceiling. In the static scenario, the male test person is sitting down the entire time, emulating the behavior of a normal person who is working at his desk. Measurement results, presented in Table 3, show that the ambient-light photovoltaic cell harvested an average power of 171.3 µW in this scenario. In the second and third scenario, the male test person is walking at a normal pace the whole time. The Kinetron MGS 32.8 harvested an average power of 87.8 µW from only body movement in the second scenario. Measurement results in the hybrid scenario show that the system harvested up to 229.8 µW when worn on a person’s wrist in an illuminated room.

**Table 3:** Summary of system performance for all indoor scenarios (average harvested power $\overline{P}_{harv}$) and average total harvested energy $\overline{E}_{total}$).

	$\overline{P}_{harv}$ $(\upmu$ W)	$\overline{E}_{total,1 min}$ (mJ)	$\overline{E}_{total,1 min}$ ($\upmu$ Wh)
Ambient-light scenario	171.3	10.3	2.9
Body movement scenario	87.8	5.3	1.5
Hybrid scenario	229.8	13.8	3.8

### Representative application scenario

These very promising measurement results enable us to further develop this wearable coupled-QM SIW antenna platform for numerous applications by extending the system with sensors for monitoring the biophysical parameters of the person wearing it and/or the conditions of his/her environment. Target user groups are mainly rescue workers, military personnel, and other safety workers. Moreover, the system could be adapted to integrate into SFIT systems in the future. This motivates the choice for a protective rubber foam, such as EPDM, to unobtrusively integrate this wearable antenna platform into protective clothing. The system architecture can be easily extended with different sensors by simply connecting the LDO of the CPMS in Fig. 4 to the V_DD_ of the MCU and to the different sensors.

**Figure 11:**
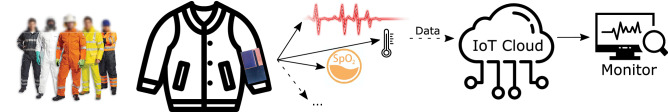
Representative application scenario: Battery-less vital signs (body temperature, blood oxygen level (SpO_2_) and heart rate) monitoring.

**Table 4:** Overview of power consumption for the proposed application with $V_{DD}$ = 3.3 V.

	Time (ms)	Current (mA)	Energy $(\upmu$ Wh)
BLE transmission	20	5.2	0.095
MAX30205	50	0.6	0.028
MAX30102	4000	0.6	2.2
STM32WB35 (16 MHz)	4000	2	7.3

An elaborated application scenario consists is monitoring the vital signs (body temperature, blood oxygen level (SpO_2_) and heart rate) of a person and report these data back to a central node, without the use of batteries (Fig. 11). Recent applications of Bluetooth-Low-Energy (BLE) sensors show a current consumption of 9 mA during approximately 20 ms for the transmission of 1 advertisement packet^41^. The wireless ULP MCU applied here, being STM32WB35, features even better current consumption specifications of 5.2 mA in Tx mode at 0 dBm^42^. The use of advertisement packets can be very useful for this application, since no system pairing is needed. This may be combined with two ULP sensors such as the MAX30205 sensor and the MAX30102 sensor for monitoring body temperature, SpO_2_ and heart rate, with a typical operating current of 600 µA for both sensors^43,44^. Sensing the body temperature of the user can be performed in only 50 ms with the MAX30205 sensor, whereas the MAX30102 sensor takes usually 4 s to perform an accurate measurement of the heart rate of the user. In a final step, the active current consumption of the STM32WB35 needs to be taken into account. In a low power mode with a clock frequency of 16 MHz, the current consumption can be reduced to 2 mA. An overview of the power consumption for the proposed application is given in Table 4. Based on the results in the hybrid scenario, it would be possible to sense and transmit the human body temperature, the blood oxygen level and the heart rate approximately every 2.5 minutes, based on the hybrid kinetic and ambient-light energy harvesting results in Table 3.

## Conclusion

In this paper, a novel technique was proposed to compactly and seamlessly integrate a micro generator system inside the substrate of a wearable coupled quarter-mode substrate-integrated-waveguide antenna. In addition, a flexible PCB with a central power management system, a low-profile supercapacitor of 15 mF and a wireless ULP microcontroller was developed to be deployed on the feed plane of the antenna. In a final step, a flexible ambient-light photovoltaic cell was deployed directly on top of the antenna radiator to enable hybrid EH. Measurements and simulations of the antenna platform showed that the completely integrated system achieves an impedance bandwidth of 307 MHz (12.5%), a realized gain of 3.74 dBi and an overall efficiency of 88.57%. The achieved bandwidth covers the entire [2.4; 2.4835] GHz frequency band and provides margins of around 100 MHz at both sides of the spectrum, as intended. Additional simulations and measurements under cylindrical bending and on-body were performed to confirm satisfactory antenna performance under realistic conditions. Next, the performance of the proposed energy harvesting and power management module was also tested in different real-life indoor scenarios, when the antenna platform is worn as a wearable sensor node around a person’s wrist. Measurement results confirm the proper operation of the proposed hybrid energy harvesting circuit. A combination of body movement and indoor light in a hybrid scenario may yield an average harvested power of 229.8 µW, which is more than sufficient to power modern wireless sensors and ULP devices.

## Data Availability

The datasets generated during and/or analyzed during the current study are available from the corresponding author on request.
